# Efficacy of adalimumab therapy in experimental rat sclerosing encapsulated peritonitis model

**DOI:** 10.3325/cmj.2019.60.431

**Published:** 2019-10

**Authors:** Yeliz Akgun, Serkan Bakirdogen, Meral Gulay Kadioglu Kocak, Sibel Bektas, Ceren Demir, Erdem Akbal, Sait Elmas

**Affiliations:** 1 Department of Internal Medicine, School of Medicine, Canakkale Onsekiz Mart University, Canakkale, Turkey; 2 Department of Nephrology, School of Medicine Canakkale Onsekiz Mart University, Canakkale, Turkey; 3 Department of Nephrology, Ministry of Health, Okmeydani Educating and Research Hospital, Istanbul, Turkey; 4 Department of Pathology, Health Sciences University School of Medicine, Gaziosmanpasa Educating and Research Hospital, Istanbul, Turkey; 5 Department of Gastroenterology, Bilim University School of Medicine, Istanbul, Turkey; 6 Experimental Medicine and Research Center, Canakkale Onsekiz Mart University Canakkale, Turkey

## Abstract

**Aim:**

To investigate the efficacy of adalimumab treatment in an experimental rat sclerosing encapsulated peritonitis (SEP) model.

**Methods:**

The study involved 40 Wistar albino rats divided into four groups: chlorhexidine (CH) group, control group, CH + adalimumab group, and CH + resting group. The control group received normal saline intraperitoneally (i.p.). Other groups received 0.1% CH gluconate, 15% ethanol, and normal saline mixture i.p. for three weeks in order to induce SEP. CH + adalimumab group received 5 mg/kg adalimumab i.p. at the beginning of week 4 and week 6, while CH + resting group was followed-up for three weeks without applying any procedure after the onset of SEP. Rats in groups CH and control group were sacrificed on day 21, and rats in group CH + adalimumab and CH + resting were sacrificed on day 42. All groups were evaluated for peritoneal thickness, inflammation, vascularization, and fibrosis.

**Results:**

CH + adalimumab group showed a significant decrease in peritoneal thickness, fibrosis score, and vascular score compared with CH group and CH + resting group.

**Conclusion:**

Adalimumab can prevent SEP development.

Sclerosing encapsulated peritonitis (SEP) is a rare complication of peritoneal dialysis, with high morbidity and mortality. The pathological findings of SEP include mesothelial denudation, increase in submesothelial thickness, interstitial fibrosis, and vasculopathy (1). Although currently there is no effective treatment for SEP, the discontinuation of peritoneal dialysis, corticosteroid and tamoxifen use, surgical treatments, and total parenteral nutrition can be applied (2). Therapies targeting vascular endothelial growth factor (VEGF), renin-angiotensin aldosterone system, or erythropoietin and immunosuppressive drugs have been tested in experimental peritoneal sclerosis models. Some of these therapies positively affected peritoneal thickness, vascularization, and fibrosis (3-7), whereas others produced no response or produced negative results (8,9).

A central role in the pathophysiology of SEP is played by transforming growth factor beta (TGF-β) (10), whose production is triggered by transmembrane TNF-α reverse signal in macrophages (11). This is why we hypothesized that adalimumab, as a monoclonal antibody against tumor necrosis factor-alpha (TNF-α) (12), could prevent SEP development. Adalimumab therapy has previously been shown to revert angiogenesis in patients with psoriasis (13), while anti-TNF therapy has led to clinical and endoscopic recovery in Crohn’s disease, both by inhibiting vascular proliferation and due to its anti-inflammatory effects (14). The aim of our study was to investigate the efficacy of adalimumab therapy in an experimental rat SEP model.

## MATERIAL AND METHODS

### Experimental animals

The study was performed in Canakkale Onsekiz Mart University Experimental Research Application and Research Center Laboratory between June and September 2016. It involved forty 6-8-month-old Wistar albino rats (20 males and 20 females) weighing between 200 and 250 g. The rats were kept in cages containing five rats under standard caging conditions at 24°C room temperature with 12 hours of dark/light cycle and standard feeding and water supply. At the end of the study, pain control was achieved before anesthesia by 5 mg/kg intramuscular (i.m.) lidocaine injection. Rats were then sacrificed by using 60 mg/kg i.m. ketamine hydrochloride injection on day 21 or on day 42, depending on the group. The rats with signs of infection and systemic reaction to adalimumab were excluded from study.

The study was approved by Canakkale Onsekiz Mart University animal ethics committee (Decision No:2016/01-03, Approval date: January 21, 2016).

### Experimental design

Experimental SEP was induced according to Ishii et al (15). A mixture of 0.1% chlorhexidine (CH) gluconate (Drogsan Medicines Inc. Balgat, Ankara), 15% ethanol, and normal saline (NS) (10 mL/kg/d) was prepared and used aseptically. Adalimumab (Humira, 40 mg/0.8 mL [AbbVie, North Chicago, IL, USA]) was injected intraperitoneally (i.p.) at a dose of 5 mg/kg after having been dissolved in 40 mL of NS (1 mg/mL) (16). To eliminate the effects of direct damage to the peritoneum by repeated injections, daily injections were performed on the lower quadrant of the abdomen with a 21-G needle, and parietal peritoneum in the upper left quadrant was used for pathological examinations.

Four groups were formed with 10 rats in each. The duration of the study was 21 days for CH and control groups and 42 days for other groups. The groups were the following:

1. CH group (group 1) received a mixture of 0.1% CH gluconate, 15% ethanol, and NS (10 mL/kg/d) i.p. every day.

2. Control group (group 2) received NS (10 mL/kg/d) i.p. every day.

3. CH + adalimumab group (group 3) received a mixture of 0.1% CH gluconate, 15% ethanol, and NS (10 mL/kg/d) i.p. every day for 21 days. Adalimumab was administered biweekly at the fourth and sixth week at a dose of 5 mg/kg.

4. CH + resting group (group 4) received a mixture of 0.1% chlorhexidine gluconate, 15% ethanol, and NS (10 mL/kg/d) i.p. every day for 21 days. During the following 21 days, no intervention was performed.

### Histopathological evaluation

All of the formaldehyde fixed parietal peritoneal tissue samples were embedded vertically in paraffin after routine tissue follow-up, and 5-mm thick sections were made. The sections were stained with hematoxylin and eosin (H&E) and Masson’s trichrome (MT) dyes. The stained sections were evaluated under light microscopy for parietal peritoneal thickness, inflammation, and fibrosis. In addition, 4-μm sections were prepared from all samples on negatively charged slides, and immunohistochemical analyses were performed with use of TGF-β1 (1/100, mouse monoclonal immunoglobulin G [IgG], Santa Cruz Biotechnology, Inc., Dallas, TX, USA), VEGF (1/100, rabbit polyclonal IgG, Spring Bioscience, Pleasanton, CA, USA), and alpha-smooth muscle actin (α-SMA, 1/100, mouse monoclonal IgG, Cell Marque, Rocklin, CA, USA) antibodies. All pathological examinations were performed by a single pathologist who was blinded to the characteristics of the groups.

To measure parietal peritoneal thickness, microscopic images obtained from H&E-stained sections were transferred to a computer using Nikon software, version 4.30 (Nikon Instruments Inc., New York, NY, USA). Parietal peritoneal thickness was measured at ten regions in micrometers, and mean thickness was recorded. Inflammation and fibrosis were semiquantitatively scored using H&E stain and MT stain, respectively (15). Inflammation score was classified as: 0 – no inflammation or the presence of isolated inflammatory cells; 1 – mild inflammation: the presence of a few scattered inflammatory cells; 2 – moderate inflammation: the presence of large numbers of inflammatory cells in small groups within one high magnification area; 3 – severe inflammation: the presence of inflammatory cells with a diffuse distribution or in large groups. Fibrosis score was classified as: 0 – no fibrosis; 1 – mild; 2 – moderate; 3 – severe fibrosis. Vascular score was obtained by counting vascular structures reacting to α-SMA immunohistochemical staining with use of 200 × magnification at ten regions for each section, and calculating their average (5).

TGF-β1 and VEGF score was obtained by counting fibroblast and macrophage cells reacting to TGF-β1 and VEGF immunohistochemical staining with use of 200 × magnification at ten regions for each section, and calculating their average (5). Vasculopathy was graded according to the degree of subendothelial hyalinization, luminal irregularity, and narrowing, as described by Williams et al (17).

### Statistical analysis

All results are expressed as median (Q1-Q3). Due to the limited number of rats in each group, non-parametric methods were used. The difference in the distribution of categorical data was analyzed with the χ^2^ test. Peritoneal thickness, inflammation, fibrosis, and vascular score; and VEGF and TGF-β1 scores were compared between the groups with the Kruskal-Wallis test. In the *post-hoc* analysis, the Mann-Whitney U test with Bonferroni correction was used. Statistical significance level was *P* < 0.008 for the Mann-Whitney U test with Bonferroni correction and *P* < 0.05 for the other tests. The analyses were performed using the SPSS, version 20.0 for Windows (IBM Corp, Armonk, NY, USA).

## RESULTS

**Figure 1:**
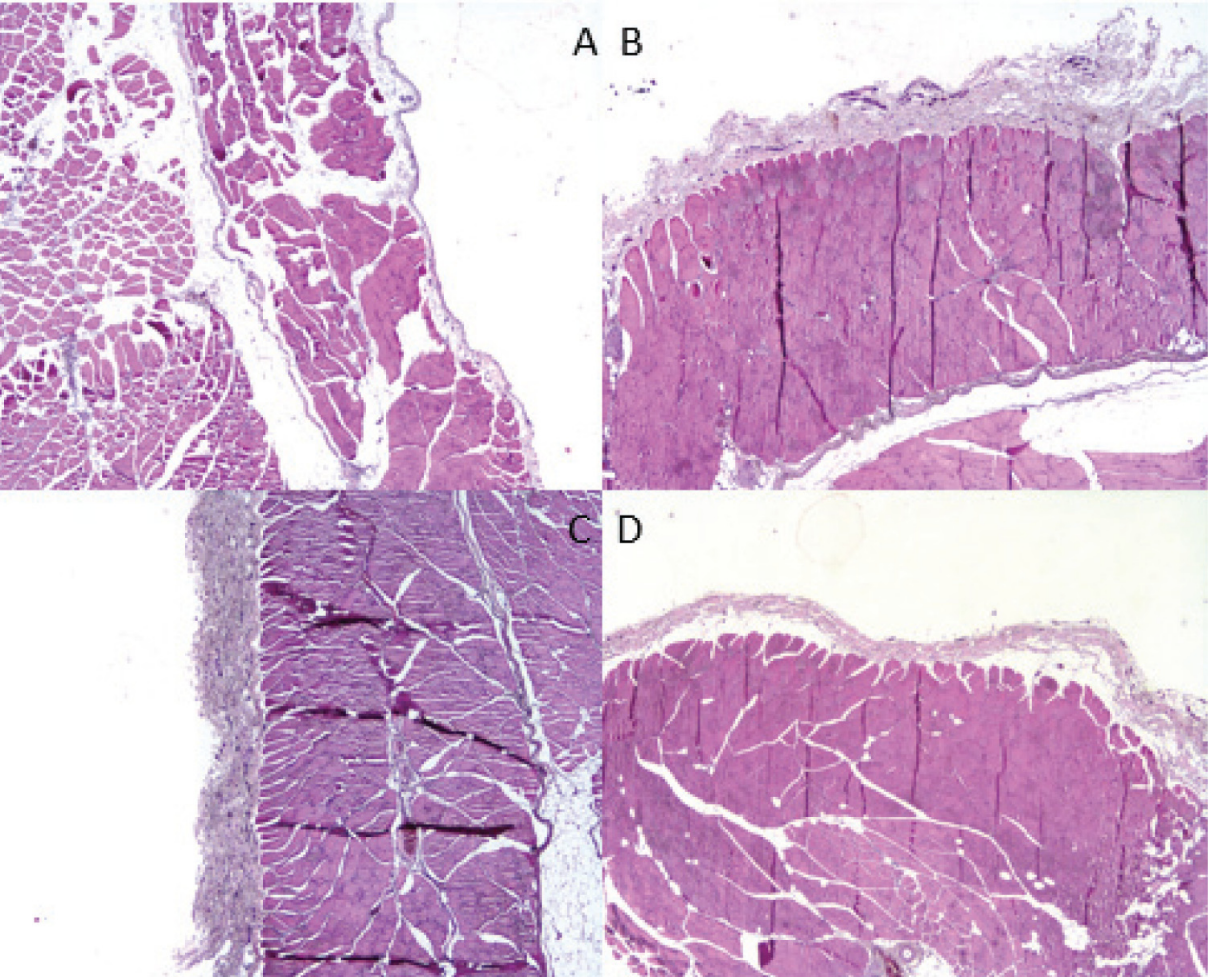
Histopathological images of the parietal peritoneum thickness (hematoxylin and eosin ×10). (**A**) Control group; (**B**) Chlorhexidine group; (**C**) Chlorhexidine + resting group; (**D**) Chlorhexidine + adalimumab group.

All 40 rats completed the study. CH group showed thickening in the parietal peritoneum and adhesion in the abdominal wall during scarification, while these changes were absent in the control group. Control group had significantly lower median peritoneal thickness than CH group, CH + resting group, and CH + adalimumab group, (*P* = 0.0001, *P* = 0.0001, and *P* = 0.003, respectively). CH + adalimumab group had significantly lower median peritoneal thickness than CH group and CH + resting group (*P* = 0.0001 and *P* = 0.0001, respectively) (Table 1 and Figure 1).

**Table 1:** Peritoneal thickness, inflammation, fibrosis, vascular score, vascular endothelial growth factor (VEGF), and transforming growth factor beta (TGF-β1) scores in different study groups

Variables, median (Q1-Q3)	Chlorhexidine group (n = 10)	Control group (n = 10)	Chlorhexidine + adalimumab group (n = 10)	Chlorhexidine + resting group (n = 10)	P*
Peritoneal thickness (μm)	394.4 (323.1-454.7)	82.8 (66.9-121.9)	181.7 (130.2-201.1)	539.8 (381.6-649.5)	0.0001
Inflammation score	1.0 (1-1)	0.0 (0-0)	1.0 (0-1)	1.5 (1-2)	0.0001
Fibrosis score	2.0 (1-2)	0.0 (0-0.5)	1.0 (1-1)	2.0 (2-3)	0.0001
Vascular score	8.0 (7-9)	0.0 (0-0)	5.0 (4.75-6)	14.8 (15-16.3)	0.0001
VEGF	5.0 (4.8-6)	0.0 (0-0)	3.0 (3-4)	7.0 (6.75-8.25)	0.0001
TGF-β1	2.0 (1.8-2)	0.0 (0-0)	1.0 (0-1)	2.0 (1.8-3)	0.0001

*Kruskal-Wallis test.

Control group had significantly lower median inflammation score than CH group, CH + resting group, and CH + adalimumab group (*P* = 0.0001, *P* = 0.0001, and *P* = 0.002, respectively). CH + adalimumab group had significantly lower median inflammation score than CH + resting group (*P* = 0.005).

Control group had significantly lower median fibrosis score than CH group, CH + resting group, and CH + adalimumab group (*P* = 0.0001, *P* = 0.0001, and *P* = 0.001, respectively). CH + adalimumab group had significantly lower median fibrosis score than CH group and CH + resting group (*P* = 0.007 and *P* = 0.001, respectively).

Control group had significantly lower median vascular score than CH group, CH + resting group, and CH + adalimumab group (*P* = 0.0001, *P* = 0.0001, and *P* = 0.0001, respectively). CH + adalimumab group had significantly lower median vascular score than CH group and CH + resting group (*P* = 0.001, *P* = 0.0001, respectively).

**Figure 2:**
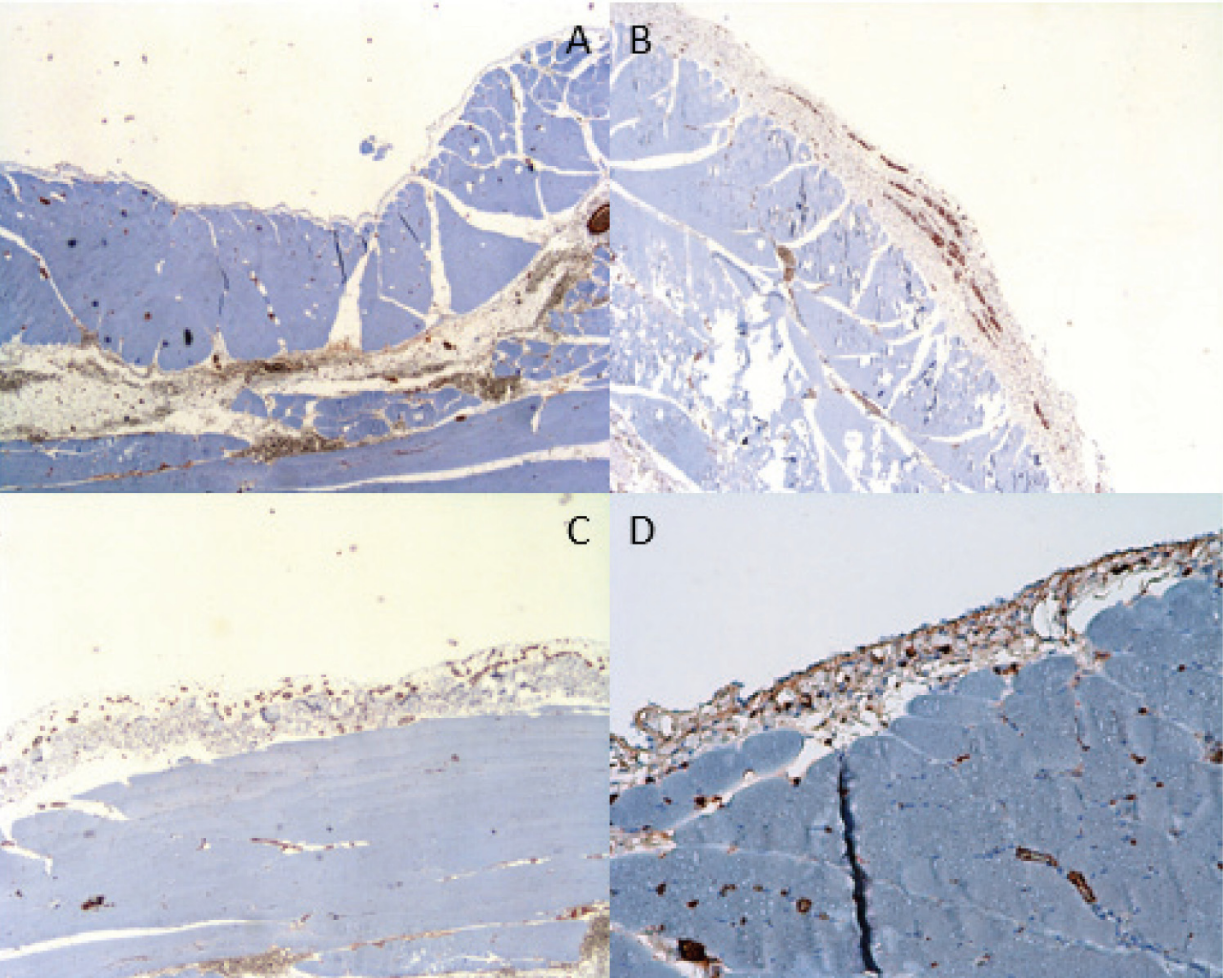
Parietal peritoneal images obtained by using vascular endothelial growth factor immunohistochemical staining. (**A**) Control group ( × 10); (**B**) Chlorhexidine group ( × 10); (**C**) Chlorhexidine + resting group ( × 10); (**D**) Chlorhexidine + adalimumab group ( × 20).

Control group had significantly lower mean VEGF score than CH group, CH + resting group, and CH + adalimumab group (*P* = 0.0001, *P* = 0.0001, and *P* = 0.0001, respectively). CH + adalimumab group had significantly lower median VEGF score than CH group and CH + resting group (*P* = 0.0001). Parietal peritoneal images obtained by using VEGF immunohistochemical staining are shown in Figure 2.

**Figure 3:**
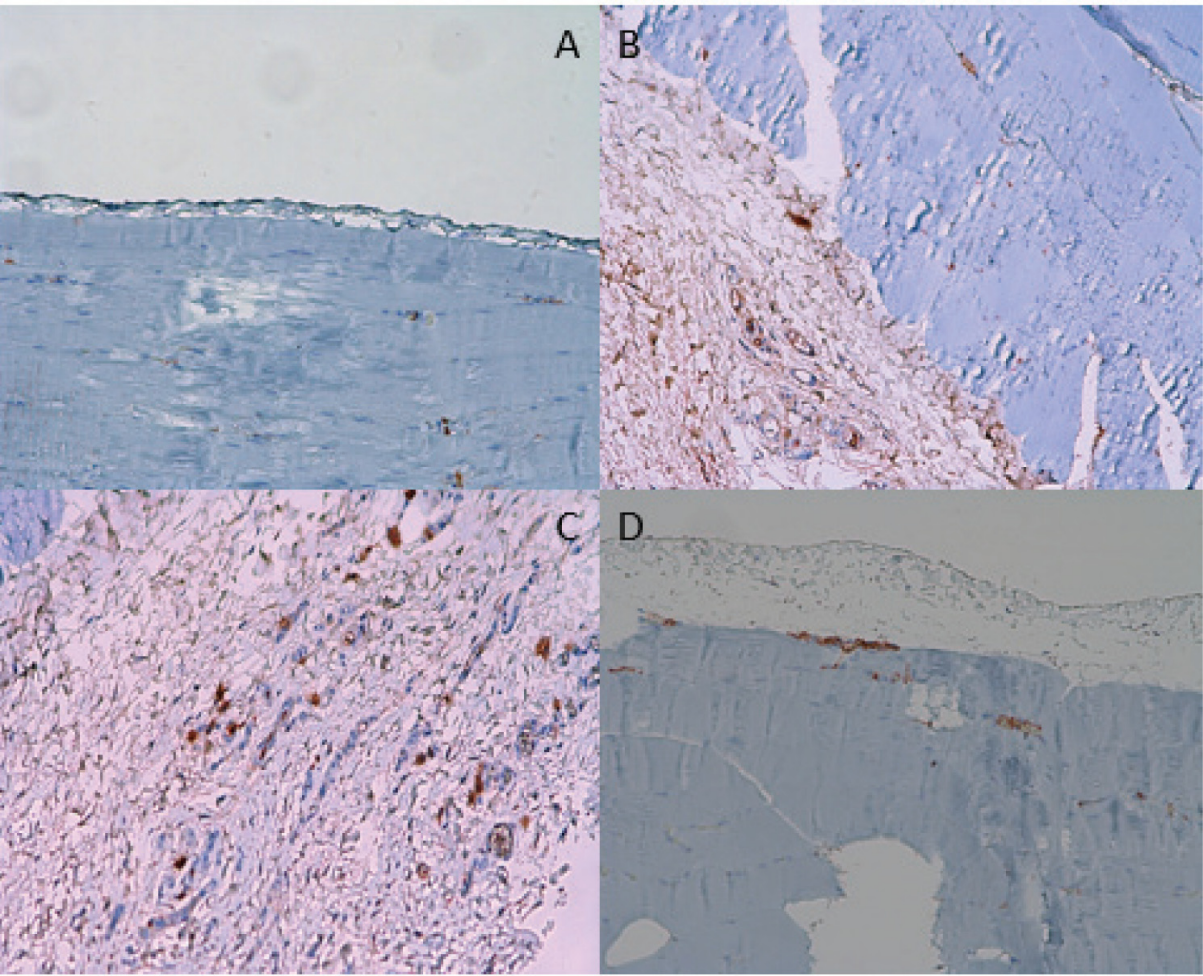
Parietal peritoneal images obtained by transforming growth factor-β1 immunohistochemical staining. (**A**) Control group ( × 10); (**B**) Chlorhexidine group ( × 10); (**C**) Chlorhexidine + resting group ( × 20); (**D**) Chlorhexidine + adalimumab group ( × 10).

Control group had significantly lower median TGF-β1 than CH group, CH + resting group, and CH + adalimumab group (*P* = 0.0001, *P* = 0.0001, and *P* = 0.002, respectively). CH + adalimumab group had significantly lower median TGF-β1 score than CH group and CH + resting group (*P* = 0.0001 and *P* = 0.001, respectively). Parietal peritoneal images obtained by TGF-β1 immunohistochemical staining are shown in Figure 3.

## DISCUSSION

Our study found that adalimumab prevented the development of experimental SEP. Although our experimental SEP model was created according to Ishii et al (15), we studied the development of peritoneal fibrosis for 3 rather than for 8 weeks. In our study, CH group had significantly higher parietal peritoneal thickness, inflammation, and fibrosis scores compared with the control group, indicating that peritoneal fibrosis was effectively induced. In CH + resting group, morphological peritoneum changes were not reversed and they even progressed, suggesting that the peritoneum was still active.

In experimental SEP models, some therapies (VEGF inhibitor, renin-angiotensin system inhibitors, aliskiren, erythropoietin, and thalidomide) reduced peritoneal thickness, vascularization, and fibrosis (3-7). However, some therapies (calcium channel blocker and cyclosporine) yielded no positive results and may even pose a risk for the development of SEP (8,9).

TNF-α acts on innate immune cells and contributes to the development of fibrosis (18). However, it also suppresses fibrosis by down-regulating connective tissue growth factor (CTGF) in fibroblasts (19). In our study, the first to use adalimumab in an experimental SEP model, CH + adalimumab treated animals had significantly lower peritoneal thickness, inflammation, fibrosis, and vascular score compared with CH group and CH + resting group.

Factors that contribute to the pathogenesis of SEP are VEGF and TGF-β (10). Human peritoneal mesothelial cells play an important role in VEGF synthesis, and VEGF release increases in the presence of proinflammatory cytokines. VEGF can increase vascular permeability, vasodilatation, and neoangiogenesis in the peritoneal membrane (20). Some therapies (aliskiren, erythropoietin, and thalidomide) applied after inducing an experimental SEP model decreased TGF-β and VEGF release (5-7). In our study, CH + adalimumab significantly decreased TGF-β1 and VEGF compared with CH and CH + resting groups, probably because of anti-TNF effects of adalimumab. Anti-inflammatory and angiogenic activity of TNF-α inhibitors has been demonstrated in the treatment of immune-associated diseases. In addition, angiogenic effects of TNF are due to changed expression of VEGF and its endothelial receptor VEGF receptor 2 (21).

Other substances that play a role in the pathogenesis of SEP are fibroblast growth factor (FGF), matrix metalloproteinase (MMP), and myeloperoxidase (10). Serum FGF and VEGF levels in psoriatic patients were decreased by anti-TNF therapy (infliximab) (22). MMP-3 was significantly reduced by the addition of adalimumab therapy in patients with rheumatoid arthritis (23) and by TNF-α antagonist therapy (infliximab, etanercept, and adalimumab) in patients with spondyloarthritis (24,25). Adalimumab therapy also reduced myeloperoxidase activity (26,27). TNF-α inhibitors suppress nuclear factor-kappaB (NF-кB) activity (28), but one study showed that adalimumab therapy was not effective on the NF-кB activity of lymphocytes in patients with rheumatoid arthritis (29).

A limitation of our study is the fact that it did not investigate immunohistochemical localization of TNF-α and CTGF in peritoneal tissue, serum FGF and MMP-3 levels, and myeloperoxidase and NF-кB activities. In addition, it did not involve groups treated with different adalimumab doses.

In conclusion, this experimental SEP model indicates that adalimumab can prevent experimental SEP development. However, more extensive experimental and clinical studies are needed to find the optimal dose and duration of treatment.
